# A Platform Approach to mRNA Purification by Tangential Flow Filtration: Application to a Pentavalent Norovirus Vaccine

**DOI:** 10.3390/vaccines14100865

**Published:** 2026-10-01

**Authors:** Emily A. Dewar, Andrew R. Swartz, Keira Tuberty, Adam Gouveia

**Affiliations:** Vaccines & Advanced Biotechnologies Process R&D, Merck & Co., Inc., Rahway, NJ 07065, USA; emily.berckman@merck.com (E.A.D.); keira.tuberty@merck.com (K.T.); agouvei2@its.jnj.com (A.G.)

**Keywords:** mRNA vaccine, norovirus, tangential flow filtration, membrane molecular weight cutoff, RNA conformation, membrane sieving, platform manufacturing

## Abstract

Background/Objectives: Multivalent mRNA vaccines are being evaluated for prevention of norovirus infection caused by multiple circulating strains. Platform purification processes may accelerate development and reduce manufacturing complexity by enabling multiple mRNA constructs to be processed using a common downstream workflow. This study aimed to develop a high-yielding tangential flow filtration (TFF) process for a pentavalent norovirus vaccine comprising five VP1-encoding mRNA constructs from genotypes GI.1, GII.2, GII.3, GII.4 and GII.6. Methods: TFF studies evaluated membrane MWCOs, transmembrane pressures, shear rates, mRNA loadings, and scale-up. Process performance was assessed based on mRNA recovery, integrity, functionality, and apparent hydrodynamic size. Results: Although the VP1 constructs had similar molecular weights (590–610 kDa), initial studies using 100 kDa membranes revealed construct-dependent losses due to permeation of full-length mRNA. Apparent hydrodynamic size was associated with observed membrane sieving behavior, suggesting that transcript-specific structural properties may influence membrane transmission. Reducing the membrane MWCO from 100 to 50 kDa eliminated detectable mRNA permeation while maintaining membrane performance. A shear rate of 4000 s^−1^ supported higher mRNA loading without compromising recovery or integrity. The resulting 50 kDa process was successfully scaled from 40 cm^2^ to 1600 cm^2^ and achieved product yields exceeding 95% across all five VP1 mRNA constructs. Conclusions: These findings support a framework for developing standardized TFF conditions for multivalent mRNA vaccines. The results indicate that transcript molecular weight and nominal membrane MWCO alone may not predict mRNA retention and demonstrate the applicability of a common TFF process across five VP1 mRNA constructs. The proposed influence of RNA conformation on membrane transmission requires confirmation using orthogonal structural methods.

## 1. Introduction

Noroviruses, members of the *Caliciviridae* family, are a leading cause of acute gastroenteritis (AGE), resulting in an estimated 21 million cases annually in the United States [1]. Clinical outcomes range from self-limiting gastrointestinal illness to severe disease and mortality in vulnerable populations, particularly young children and the elderly [2]. Noroviruses are non-enveloped icosahedral viruses containing a positive-sense single-stranded RNA genome encapsidated by 90 dimers of the major capsid protein VP1 and stabilized by the minor capsid protein VP2 [3]. Human disease is primarily associated with genogroups GI and GII, with occasional infections caused by GIV strains. The substantial genetic diversity among circulating noroviruses has complicated vaccine development and may necessitate multivalent vaccine formulations capable of eliciting immunity against multiple strains [4]. Although no norovirus vaccine has yet been approved, several VP1-based virus-like particle (VLP) and mRNA vaccine candidates are currently in clinical development [5,6]. mRNA technology is particularly attractive because antigen sequences can be rapidly modified while preserving a common manufacturing platform, enabling efficient adaptation to evolving viral strains and emerging outbreaks [7,8].

mRNA vaccine constructs are typically synthesized by in vitro transcription (IVT) from a DNA template containing a 5′ cap, untranslated regions (UTRs), a coding open reading frame (ORF), and a 3′ poly(A) tail [9]. In addition to the desired full-length transcript, IVT reactions generate process-related impurities including double-stranded RNA (dsRNA) and truncated RNA species [10]. Unlike globular proteins, mRNAs fold into sequence-dependent secondary and tertiary structures through intramolecular base pairing [11]. Consequently, transcripts with similar lengths and molecular weights may adopt more compact or extended conformations depending on their nucleotide sequence, resulting in different effective hydrodynamic dimensions [12]. These structural differences may influence transport and retention during purification operations that rely on size-dependent separation.

Following IVT, mRNA is purified to remove reaction components and process-related impurities prior to lipid nanoparticle encapsulation and formulation into the final vaccine product [13]. Common purification operations include oligo-dT affinity chromatography, hydrophobic interaction chromatography, normal-flow filtration, and tangential flow filtration (TFF) [14]. TFF is widely used for concentration and buffer exchange, where solutes passing through the membrane are collected in the permeate while retained species remain in the retentate. Membrane transport is influenced by membrane molecular weight cutoff (MWCO), transmembrane pressure (TMP), crossflow rate, and other operating conditions [15]. For protein purification by TFF, membrane MWCOs are commonly selected at least three-fold smaller than the molecular weight of the target molecule to minimize product transmission [15]. However, RNA transport through ultrafiltration membranes depends not only on molecular weight but also on molecular conformation and effective hydrodynamic size [16,17]. Consequently, full-length mRNAs may exhibit different sieving behavior despite having similar molecular weights and greatly exceeding the nominal membrane MWCO. In addition, prolonged exposure to elevated shear forces within the TFF membrane, flow path, and pump can contribute to product degradation and should be controlled during process development [18].

Despite the growing use of mRNA therapeutics and vaccines, relatively few studies have evaluated whether a single TFF operation can be applied across multiple mRNA constructs without extensive process modification. The objective of this work was to develop a platform TFF operation for the purification of a pentavalent norovirus mRNA vaccine comprising five VP1 constructs with similar molecular weights but varying coding sequences, exhibiting pairwise nucleotide identities ranging from approximately 67% to 83% (Table 1; Figure 1). Flux/TMP excursion and sieving analyses were performed to investigate factors governing mRNA transmission and product retention, including membrane molecular weight cutoff (MWCO), transmembrane pressure (TMP), and mRNA concentration. Shear rate and membrane loading were subsequently optimized to improve productivity while maintaining mRNA yield and integrity. The resulting process was then demonstrated at the pilot scale to evaluate scalability and robustness.

## 2. Materials and Methods

### 2.1. Materials

Diafiltration buffer (DF buffer, 1 mM sodium citrate, pH 6.4) was prepared using RNase-free water and 1 M sodium citrate stock solution (Teknova, Hollister, CA, USA). Sodium hydroxide used for membrane conditioning (0.1 M NaOH) was purchased from Fisher Chemical™ (Pittsburgh, PA, USA). Spectrum^®^ modified polyethersulfone (mPES) hollow fiber ultrafiltration membranes with nominal MWCOs of 50 and 100 kDa, effective lengths of 41.5 cm, and fiber inner radii of 0.25 mm (Catalog Nos. C04-E050-05-S, C04-E100-05-S, and S04-E050-05-S) were obtained from Repligen Corporation (Waltham, MA, USA). Peristaltic pumps and Masterflex tubing were purchased from Antylia Scientific (Vernon Hills, IL, USA). Pressure sensors and monitors were purchased from SciLog, Inc. (Middleton, WI, USA).

### 2.2. mRNA Synthesis and Initial Purification Process

Six mRNA vaccine constructs sharing identical non-coding elements, including the 5′ cap, untranslated regions (UTRs), and poly(A) tail, but differing in coding open reading frame (ORF), nucleotide length, and molecular weight were evaluated across multiple TFF experiments (Table 1, Figure 1). ORF sequences were aligned using the MUSCLE multiple-sequence alignment algorithm implemented in Geneious Prime (Version 2025.2.2). For sequence visualization, aligned positions were classified as matching the alignment consensus, differing from the consensus, or representing an alignment gap. Pairwise nucleotide identity and GC content were determined from the resulting sequence alignment. Predicted minimum free energy (MFE) values were calculated using RNAfold (ViennaRNA v2.7.2) and normalized to 100 nucleotides to facilitate comparison among constructs of different lengths.

mRNA was synthesized by in vitro transcription (IVT) using a protocol adapted from Henderson et al. [19]. Briefly, linearized DNA templates encoding a poly(A) tail of approximately 80 nucleotides were combined with NTPs, including N1-methylpseudouridine triphosphate in place of UTP, a 5′ cap analogue (CleanCap^®^, TriLink BioTechnologies), San Diego, CA, USA), RNase inhibitor, inorganic pyrophosphatase, and T7 RNA polymerase in a transcription buffer and incubated at 37 °C for 3 h. The IVT reaction was quenched with DNase I and incubated at 37 °C for 15 min. The quenched IVT product was diluted to 0.3 mg/mL in binding buffer (10 mM Tris, 200–800 mM NaCl, 2 mM EDTA, pH 7.0) and purified using CIMmultus^®^ Oligo dT18 (C12 Linker) (2 µm) monolithic columns (Sartorius, Göttingen, Germany) as described by Dewar et al. [20]. The mRNA was eluted from the monolithic columns using elution buffer (10 mM Tris, pH 7.0) at an RNA concentration of approximately 0.3–0.6 mg/mL to generate the TFF Load. A final TFF product mRNA concentration of 1–2 mg/mL was targeted following concentration, diafiltration, and post-use flush. Each VP1 mRNA construct was processed individually through TFF to generate drug substance and subsequently formulated into the combined pentavalent vaccine drug product. Each TFF condition was evaluated in a single run using material generated from an independent IVT reaction and chromatographic purification. All TFF experiments were performed at room temperature.

### 2.3. Evaluation of mRNA Purification with 100 kDa Hollow Fiber TFFs

Initial TFF experiments were performed using 100 kDa hollow fiber systems assembled with recirculation and permeate reservoirs, peristaltic pumps, Masterflex tubing, pressure gauges, and a retentate clamp. The TFF crossflow rate setpoint was calculated based on the target wall shear rate using Equation (1):

(1) $$\gamma =\frac{4Q}{\mathrm{N\pi}r^{3}}$$

where *γ* is the wall shear rate, *Q* is the crossflow rate, *N* is the number of hollow fibers, and *r* is the fiber inner radius. The 40 cm^2^ membranes evaluated had 6 fibers, while the 1600 cm^2^ membranes had 250 fibers.

Membranes were conditioned with 10 L/m^2^ each of water, 0.1 M NaOH, and diafiltration buffer prior to use. Each hollow-fiber membrane was used for a single TFF experiment and discarded after completion of the experiment. TFF Load material was recirculated at the target operating conditions for >5 min before concentration to an 8-fold volume concentration factor (VCF) under constant transmembrane pressure (TMP) control at approximately 2 psig. TMP was calculated as the average of the feed and retentate pressures minus the permeate pressure.

The concentrated material was subsequently diafiltered against 6 diavolumes (DV) of diafiltration buffer while maintaining the target TMP. Permeate generated during concentration and diafiltration was collected in a common permeate reservoir. TMP and permeate flux were continuously monitored throughout the TFF operation. Following diafiltration, the product was recovered and the hollow fiber was flushed in reverse flow with diafiltration buffer for 10 min. The flush fraction was pooled with the recovered material to generate the final TFF Product. Samples of the TFF Load, TFF Product, and pooled permeate were analyzed by UV absorbance at 260 nm to determine total RNA concentration and estimate RNA mass balance, according to Equations (2)–(4).

(2) $$\mathrm{TFF}\mathrm{Product}\mathrm{Yield}=\frac{\left(C_{\mathrm{product}}\right)\left(V_{\mathrm{product}}\right)}{\left(C_{\mathrm{load}}\right)\left(V_{\mathrm{load}}\right)}\times 100\%$$

(3) $$\mathrm{Permeate}\;\mathrm{Loss}=\frac{\left(C_{\mathrm{permeate}}\right)\left(V_{\mathrm{permeate}}\right)}{\left(C_{\mathrm{load}}\right)\left(V_{\mathrm{load}}\right)}\times 100\%$$

*System Loss* = 100% − (*C_product_*) (*V_product_*) − (*C_permeate_*) (*V_permeate_*) (4)

where *C_load_*, *C_product_*, and *C_permeate_* are the mRNA concentrations (mg/mL) of the TFF Load, TFF Product, and pooled permeate fractions, respectively, and *V_load_*, *V_product_*, and *V_permeate_* are the corresponding sample volumes (mL). To estimate RNA sieving over the complete TFF operation, the measured RNA Loss to Permeate was fit to Equation (5) to determine the average observed sieving coefficient (*S_ave_*).

(5) $$\mathrm{RNA}\;\mathrm{Loss}\;\mathrm{to}\;\mathrm{Peremate}=\left(1-e^{\left({-S}_{\mathrm{ave}}\left(\ln\mathrm{VCF}+\mathrm{DV}\right)\right)}\right)\times 100\%$$

where *S_ave_* is the average observed sieving coefficient, *VCF* is the volume concentration factor, and *DV* is the number of diavolumes. TFF Product samples were analyzed by agarose gel electrophoresis and dynamic light scattering (DLS).

### 2.4. Permeate Flux Excursion and mRNA Sieving Analysis

Flux excursion experiments were performed using GII.6 mRNA and 50 kDa or 100 kDa hollow fibers following the method described by Agrawal et al. [15]. TFF systems were operated at the target crossflow rate corresponding to the desired shear rate, while a retentate pinch clamp was adjusted to achieve the target transmembrane pressure (TMP). After equilibration for at least 5 min at each TMP setpoint, permeate was collected over a defined time interval and permeate flux (*J_P_*) was calculated according to Equation (6).

(6) $$J_{P}\left(\mathrm{LMH}\right)=\frac{\mathrm{Vp}\times 0.06}{T\times A}$$

where *V_P_* is the permeate volume (mL), *T* is the collection time (min), and *A* is the membrane area (m^2^). Flux excursion studies were conducted at both the initial mRNA concentration and following concentration to the target volume concentration factor (VCF). Retentate and permeate samples were collected after equilibration at each TMP setpoint and analyzed by UV absorbance at 260 nm to determine mRNA concentration. The observed sieving coefficient (*S_obs_*) was calculated according to Equation (7).

(7) $$S_{\mathrm{obs}}=\frac{C_{\mathrm{permeate}}}{C_{\mathrm{retentate}}}$$

where *C_permeate_* and *C_retentate_* are the mRNA concentrations (mg/mL) measured in the permeate and retentate samples, respectively. Selected retentate and permeate samples were further analyzed by capillary electrophoresis (CE) to assess mRNA integrity.

### 2.5. Shear Rate and mRNA Loading Studies

The effects of shear rate and membrane loading were evaluated using GII.4 mRNA and 50 kDa hollow fibers following the standard TFF operation described in Section 2.3, consisting of approximately 8-fold concentration and 6 DV diafiltration. Crossflow rates were adjusted to target shear rates of 2000, 4000, and 6000 s^−1^ at a membrane loading of 10 g/m^2^. Membrane loading was evaluated at a shear rate of 4000 s^−1^ using loadings of 10, 15, and 20 g/m^2^. TFF Product Yield was calculated using Equation (2), and relative purity was determined by capillary electrophoresis (CE) according to Equation (8).

(8) $$\mathrm{Relative}\mathrm{Purity}=\frac{{\left\{\mathrm{MP}\right\}}_{\mathrm{Product}}\left(\%\right)}{{\left\{\mathrm{MP}\right\}}_{\mathrm{Load}}\left(\%\right)}\times 100\%$$

where {*MP*}*_Product_* and {*MP*}*_Load_* are the CE main peak percentages of the TFF Product and TFF Load, respectively. The average diafiltration flux was calculated as the arithmetic mean of all permeate flux measurements collected during diafiltration.

### 2.6. Pilot Scale TFF Demonstration

The optimized TFF process was evaluated at pilot scale using GII.4 mRNA and 50 kDa hollow fiber systems with membrane areas of 40 cm^2^ and 1600 cm^2^ operated at a loading of 12–15 g/m^2^, TMP of 3–4 psig, and a shear rate of 4000 s^−1^. TFF Product Yield, relative purity, and product integrity by agarose gel electrophoresis were evaluated. Membrane permeability was calculated according to Equation (9).

(9) $$\mathrm{Permeability}=\frac{J_{p}}{\mathrm{TMP}}$$

where *J_p_* is the permeate flux (LMH) and TMP is the transmembrane pressure (psig) at each recorded timepoint. Additional pilot-scale TFF purification runs were performed using all five VP1 mRNA constructs and 1600 cm^2^, 50 kDa hollow-fiber membranes operated at a loading of 8–9 g/m^2^, TMP of ~3 psig, and a shear rate of 4000 s^−1^. TFF product yield and relative purity were determined for each construct.

### 2.7. mRNA Concentration by UV-Vis Spectrophotometry

Absorbance at 260 nm was measured using a Stunner instrument (Unchained Labs, Pleasanton, CA, USA) or a NanoDrop 8000 spectrophotometer (Thermo Fisher Scientific, Waltham, MA, USA). Total RNA concentration was calculated according to Equation (10) using an RNA-specific extinction coefficient of 40 µg/mL per absorbance unit at 260 nm.

(10) $$\mathrm{Concentration}\left(\frac{\mathrm{mg}}{\mathrm{mL}}\right)=A260\;(\mathrm{AU})\times \mathrm{Dilution}\mathrm{Factor}\times 0.04\left(\frac{\mathrm{mg}}{\mathrm{AU}-\mathrm{mL}}\right)$$

where A260 is the absorbance measured at 260 nm and Dilution Factor is the sample dilution factor.

### 2.8. Analysis of mRNA Integrity by Capillary Electrophoresis

mRNA integrity was evaluated using a microchip capillary electrophoresis method described by Raffaele et al. [21]. Samples were diluted to 10 µg/mL using Brij^®^ 58 (MilliporeSigma, Burlington, MA, USA), formamide, and 10× sample buffer supplied in the RNA reagent kit (Catalog No. CLS960010, PerkinElmer, Waltham, MA, USA). Diluted samples were heated at 70 °C for 10 min, cooled on ice for at least 5 min, and analyzed using a LabChip^®^ GXII Touch instrument (Revvity, Waltham, MA, USA). Fluorescence electropherograms were processed using Empower 3 software (Waters, Milford, MA, USA). The percentage of the main peak relative to the total electropherogram area was used as a measure of mRNA integrity and relative purity. All permeate samples were concentrated using 10 kDa Vivaspin centrifugal devices (Cytiva) before CE analysis because the RNA concentrations were below the assay target concentration. CE was used to quantify the relative proportions of the main mRNA peak and lower-molecular-weight fragments in the concentrated permeate samples.

### 2.9. Analysis of mRNA Hydrodynamic Diameter by DLS

Apparent hydrodynamic diameter of purified mRNA samples was measured by dynamic light scattering (DLS) using a Zetasizer Ultra particle analyzer (Malvern Panalytical, Malvern, UK). Samples (100 µL) were loaded into disposable low-volume cuvettes (ZEN0040, Malvern Panalytical) and equilibrated for 60 s prior to analysis. Measurements were performed at 25 °C using automatic instrument settings in triplicate with a dispersant refractive index of 1.330. Z-average diameter (d.nm) and polydispersity index (PDI) were calculated using the General Purpose analysis model in Zetasizer software (v4.1.0, Malvern Panalytical). Reported DLS values represent the mean of three technical measurements of each sample.

### 2.10. Analysis of mRNA Translatability by Cell-Free Translation

Cell-free translation (CFT) was performed following a published procedure by Stiving et al. [22]. mRNA was added to a CFT reagent mixture and incubated in a heat block at 25 °C for 2 h. After 2 h, the CFT mixture was aliquoted for subsequent analysis by Simple Western™ capillary western blot (Protein Simple, San Jose, CA, USA) following the manufacture’s procedure to quantitate translated protein content relative to a reference standard using a cross-reactive VP1 protein primary detection antibodies. Results were analyzed in Compass software (v6.1, Protein Simple) and were reported as a relative chemiluminescent signal of a test sample to the corresponding strain-specific VP1 reference standard.

## 3. Results

### 3.1. TFF Purification of 6 mRNA Constructs with 100 kDa Membranes

Six unique mRNA constructs were purified using a TFF process consisting of an 8-fold concentration step followed by 6 DV of diafiltration. Initial operating conditions were adapted from a previously established TFF process used for the purification of a coronavirus spike mRNA construct, which demonstrated high product recovery using 100 kDa hollow fibers. To minimize mRNA degradation and product loss, the process was operated at a low shear rate (2000 s^−1^), low transmembrane pressure (2–3 psig), and low membrane loading (4 g/m^2^). The 100 kDa hollow fibers were retained for the norovirus constructs because their nominal MWCO remained approximately six-fold smaller than the molecular weight of the smallest construct evaluated (~590 kDa; Table 1) and therefore were expected to provide high product retention. Under these conditions, TFF Product relative purity remained approximately 100% for all constructs, indicating no detectable product fragmentation during processing (Figure 2A). Agarose gel electrophoresis likewise demonstrated intact full-length transcripts following TFF purification (Figure 2C). In addition, cell-free translation activity was maintained across the TFF operation, indicating that TFF processing did not adversely affect mRNA function (Figure 2A).

Despite applying TFF conditions previously shown to provide high recovery of the coronavirus spike mRNA construct, substantial differences in mRNA recovery were observed among the norovirus constructs. The coronavirus spike construct (~1210 kDa), which was originally used to establish the TFF operating conditions, achieved greater than 95% TFF product yield with negligible RNA loss to permeate (Figure 2A). In contrast, the five norovirus mRNAs exhibited construct-dependent recovery despite sharing similar molecular weights (590–610 kDa), GC contents (64.2–66.1%), and predicted folding energies (−39.5 to −42.5 kcal/mol/100 nt; Table 1). Among the norovirus constructs, GII.3 achieved greater than 90% recovery, whereas GII.6 exhibited the lowest TFF product yield (~70%). GII.4, GI.1, and GII.2 exhibited intermediate recoveries ranging from approximately 80–90% (Figure 2A). Corresponding mass balances demonstrated that reduced product recovery was primarily associated with RNA loss to permeate, with smaller contributions from system loss that may reflect mRNA retained within the TFF system or adsorbed to membrane and flow-path surfaces.

To further evaluate construct-dependent membrane transmission, measured RNA loss to permeate was fit to Equation (4) to estimate the average observed sieving coefficient (*S_ave_*). Average observed sieving coefficients ranged from approximately 0.008 to 0.03 for GII.4, GII.6, GI.1, and GII.2, whereas no measurable sieving was observed for GII.3 or coronavirus spike (Figure 2B). While these values appear small, the cumulative impact over an 8-fold concentration and 6-DV diafiltration operation resulted in substantial product loss. Comparison with Table 1 showed no obvious relationship between membrane transmission and molecular weight, GC content, or predicted minimum free energy. All five norovirus constructs exhibited similar molecular weights (590–610 kDa), GC contents (64.2–66.1%), and normalized predicted MFEs (−39.5 to −42.5 kcal/mol/100 nt), yet their RNA loss to permeate ranged from undetectable for GII.3 to approximately 18% for GII.6 with potential additional losses to the TFF system.

Dynamic light scattering (DLS) measurements revealed differences in apparent hydrodynamic diameter among the norovirus mRNA constructs (Figure 2D). GII.6 and GII.2, which exhibited the greatest RNA loss to permeate, had the smallest measured Z-average diameters (~45 nm), whereas GII.3 exhibited the largest apparent diameter (~60 nm) and no measurable membrane transmission. Intermediate diameters were observed for GII.4 and GI.1, which also exhibited intermediate levels of RNA loss to permeate. All constructs exhibited relatively broad size distributions (PDI 0.2–0.4), indicating substantial polydispersity and limiting interpretation of the absolute Z-average values. However, the observed trend between apparent hydrodynamic diameter and membrane transmission suggested that differences in RNA size distribution may contribute to construct-dependent sieving behavior.

### 3.2. Evaluation of mRNA Sieving with 50 kDa and 100 kDa Hollow Fibers

To investigate the unexpected construct-dependent permeation observed with 100 kDa hollow fibers, additional studies were performed using GII.6 mRNA, which exhibited the lowest TFF product yield and highest estimated average sieving coefficient in Figure 2. Flux excursion experiments were conducted using 50 kDa and 100 kDa hollow fibers at both the initial mRNA concentration (~0.5 mg/mL) and following concentration to approximately 2 mg/mL. Permeate flux increased with increasing TMP for both membrane MWCOs and mRNA concentrations (Figure 3A). At equivalent TMP values, 100 kDa hollow fibers exhibited higher permeate flux than 50 kDa hollow fibers. Increasing mRNA concentration from ~0.5 mg/mL to ~2 mg/mL reduced permeate flux for both membranes, consistent with increased concentration polarization and reduced membrane permeability during concentration.

In contrast to the modest differences observed in permeate flux, membrane MWCO and TMP had pronounced effects on mRNA sieving behavior (Figure 3B). No measurable sieving was observed with 50 kDa hollow fibers across the evaluated TMP range at either mRNA concentration. However, substantial mRNA transmission was observed with 100 kDa hollow fibers. At the initial mRNA concentration (~0.5 mg/mL), the observed sieving coefficient increased from approximately 0.07 at 1 psig TMP to greater than 0.20 at 4 psig TMP. Following concentration to ~2 mg/mL, lower sieving coefficients were observed at comparable TMP values, although measurable mRNA transmission remained evident at elevated TMP. These results indicate that membrane transmission was strongly dependent on both MWCO and TMP, while increasing mRNA concentration reduced but did not eliminate permeation through the 100 kDa membrane.

Capillary electrophoresis analysis of selected permeate samples showed that the main peak corresponding to full-length mRNA represented the predominant RNA species, with only minor fragment peaks detected (Figure 3C). Analysis of additional permeate samples from other constructs similarly demonstrated sieving of full-length mRNA. Electropherograms collected from permeate samples generated at both low and high TMP exhibited a dominant peak corresponding to the full-length GII.6 transcript that closely matched the TFF Product sample. Minimal RNA fragments were detected in any sample, and the electropherogram profiles remained largely unchanged between product and permeate fractions. These results demonstrate that the yield losses observed with 100 kDa hollow fibers resulted from the permeation of full-length mRNA rather than fragmented species. Furthermore, reducing the membrane MWCO from 100 kDa to 50 kDa eliminated detectable permeation while maintaining comparable flux performance.

### 3.3. Optimization of Shear Rate and mRNA Loading Using 50 kDa TFFs

Because reducing the membrane MWCO from 100 kDa to 50 kDa eliminated detectable mRNA permeation without substantially affecting flux performance (Figure 3), subsequent studies focused on optimization of shear rate and membrane loading using GII.4 mRNA. Increasing shear rate from 2000 to 6000 s^−1^ had minimal impact on either TFF product yield or relative purity, both of which remained approximately 100% across the evaluated range (Figure 4A). These results indicate that no detectable mRNA degradation or fragmentation occurred over the shear rate range tested. In contrast, average diafiltration flux increased substantially with increasing shear rate, rising from approximately 18 LMH at 2000 s^−1^ to approximately 40 LMH at 6000 s^−1^. A shear rate of 4000 s^−1^ was selected for subsequent studies because it provided a significant improvement in flux relative to 2000 s^−1^ while balancing the increased pump demand required to achieve the higher crossflow rates.

Membrane loading was subsequently evaluated at 10, 15, and 20 g/m^2^ using a shear rate of 4000 s^−1^ (Figure 4B). TFF product yield and relative purity remained approximately 100% across the evaluated loading range, indicating no adverse effect of increased loading on mRNA recovery or product quality. Average diafiltration flux also remained relatively constant, ranging from approximately 30 to 35 LMH. Together with the shear rate studies, these results established a broad operating window for the 50 kDa process and supported the selection of a 4000 s^−1^ shear rate and membrane loadings up to 20 g/m^2^ for subsequent pilot-scale evaluation.

### 3.4. Pilot Scale-Up Demonstration Using Selected TFF Parameters

The optimized TFF process was evaluated by a 40-fold scale increase in membrane area from 40 cm^2^ to 1600 cm^2^ while maintaining a shear rate of 4000 s^−1^ and comparable membrane loading (12–15 g/m^2^). Membrane permeability profiles were highly similar between scales throughout both concentration and diafiltration, decreasing during concentration as mRNA concentration increased and subsequently stabilizing during diafiltration (Figure 5A). The comparable permeability profiles indicate that membrane performance and concentration polarization behavior were maintained following scale-up.

Process performance was also comparable between scales (Table 2). Average diafiltration fluxes were similar, with values of 31.0 and 30.7 LMH observed for the 40 cm^2^ and 1600 cm^2^ scales, respectively. TFF product yield remained high at both scales (97–98%), while mRNA integrity and functionality were maintained across the operation. Agarose gel electrophoresis similarly demonstrated no detectable differences in mRNA integrity between the lab-scale and pilot-scale TFF products (Figure 5B), and the cell-free translation assay confirmed that the pilot-scale mRNA remained functional following TFF processing (Table 2). These results demonstrate successful scale-up of the optimized 50 kDa TFF process while maintaining membrane performance, product recovery, and mRNA integrity.

To evaluate the broader applicability of the selected operating conditions, pilot-scale TFF runs were performed for each VP1 mRNA construct using 1600 cm^2^, 50 kDa hollow-fiber membranes operated at a TMP of ~3 psig and a shear rate of 4000 s^−1^. Product yields exceeded 95% for all five constructs, while relative mRNA purity remained comparable across runs (Figure 5C). No RNA was detected in the permeate across any of the runs, demonstrating complete retention of the mRNA product by the 50 kDa membrane. These findings indicate that the platformed 50 kDa TFF process can be applied to the purification of all five VP1 mRNA constructs.

## 4. Discussion

In this work, a TFF process was evaluated using six mRNA vaccine constructs ranging from approximately 600 to 1200 kDa, including five norovirus VP1 mRNAs intended for use in a pentavalent vaccine formulation. Initial studies using 100 kDa hollow fibers revealed unexpected construct-dependent loss of mRNA to the permeate. GII.4, GII.6, GI.1 and GII.2 exhibited measurable sieving through the membrane, whereas GII.3 and coronavirus spike did not, despite all constructs having molecular weights approximately six-fold greater than the membrane MWCO (Figure 2 and Figure 3). Furthermore, mRNA integrity and cell-free translation functionality were maintained across the TFF operation. Capillary electrophoresis confirmed that the RNA detected in the permeate consisted primarily of full-length mRNA rather than fragmented species, indicating that yield losses resulted from sieving of intact transcripts rather than degradation during processing. The five norovirus constructs exhibited different membrane sieving behavior despite possessing similar molecular weights, GC contents, and predicted folding energies. Multiple-sequence alignment revealed nucleotide identities ranging from 67–83%, with sequence differences distributed throughout the VP1 coding regions rather than localized to a specific region (Figure 1A,B). Average observed sieving coefficients ranged from undetectable for GII.3 to approximately 0.03 for GII.6 (Figure 2B), whereas the corresponding molecular weights differed by less than 5% (Table 1).

These observations indicate that RNA membrane sieving behavior could not be predicted by conventional sequence descriptors. Previous studies have similarly reported that nucleic acid transport through ultrafiltration membranes depends on effective molecular size and conformational flexibility rather than molecular weight alone [17]. Nucleic acids adopt secondary and tertiary structures through intramolecular base pairing that can result in conformations ranging from relatively compact to more extended structures [23]. Comparison of the multiple-sequence alignment, pairwise nucleotide identity, GC content, and predicted MFE with the observed sieving coefficients did not identify a sequence-level descriptor that explained the construct-dependent differences in membrane transmission **(**Figure 1A,B; Table 1). Consequently, the effective size of an RNA molecule presented to the membrane may differ substantially from that predicted by molecular weight alone, leading to differences in observed sieving behavior.

Experimental evidence supporting this concept has been reported for DNA plasmids and smaller RNA molecules [24,25]. Manzano et al. demonstrated that a 70-nt hairpin RNA exhibited greater transmission through an ultrafiltration membrane than a linear RNA of identical molecular weight, consistent with its smaller hydrodynamic size measured by dynamic light scattering. In addition to hydrodynamic size, RNA conformation may alter the presentation of the negatively charged phosphate backbone, thereby influencing electrostatic interactions with membrane surfaces [16].

DLS measurement showed an association between apparent hydrodynamic size and membrane sieving behavior. Constructs exhibiting the highest membrane transmission, GII.6 and GII.2, also exhibited the smallest apparent hydrodynamic diameters, whereas GII.3 exhibited the largest apparent diameter and no measurable transmission (Figure 2D). However, all constructs exhibited relatively broad size distributions (PDI 0.2–0.4), indicating heterogeneous RNA populations. Because the reported Z-average diameter represents an intensity-weighted average of the measured distribution, the interpretation of DLS results with respect to specific RNA conformations is limited for these polydisperse samples [26]. While the DLS measurements suggest differences in apparent hydrodynamic size among the constructs, the observed association does not establish a causal relationship between RNA conformation and membrane transmission. Orthogonal structural characterization and computational modeling [27] are needed to determine how RNA conformation influences membrane sieving behavior.

These results demonstrate that molecular weight alone was insufficient to predict membrane retention for these mRNA constructs. Measurable sieving was observed even when mRNA molecular weights exceeded the nominal membrane MWCO by approximately six-fold, indicating that membrane selection criteria commonly applied to proteins [15] may not ensure complete retention of structurally diverse mRNAs. Reducing the membrane MWCO from 100 kDa to 50 kDa eliminated detectable product permeation while maintaining comparable permeability and flux performance (Figure 3). These findings suggest that membrane MWCOs greater than ten-fold lower than the target mRNA molecular weight may provide a more conservative approach for minimizing construct-dependent sieving.

Previous studies have recommended minimizing shear exposure to reduce the risk of nucleic acid degradation during TFF processing [17,28]. However, no reduction in product yield or relative purity was observed when shear rate was increased from 2000 to 6000 s^−1^ (Figure 4). In addition, higher shear rates improved diafiltration flux and could enable faster processing times. These results indicate that the mRNA constructs evaluated in this study were not adversely affected by the shear rates examined and that operation at 4000 s^−1^ provided a practical balance between flux performance and pump demand during scale-up.

Membrane loading up to 20 g/m^2^ had no measurable impact on product recovery, relative purity, or flux performance (Figure 4). Based on these findings, a 50 kDa membrane and a 4000 s^−1^ shear rate were selected for pilot-scale evaluation. The optimized process was successfully scaled from 40 cm^2^ to 1600 cm^2^ membrane area using GII.4 mRNA at membrane loadings of 12–15 g/m^2^ while maintaining comparable permeability profiles, product recovery, and mRNA integrity (Figure 5A,B; Table 2). Pilot-scale TFF runs using all five VP1 mRNA constructs at membrane loadings of 8–9 g/m^2^ resulted in product yields exceeding 95% and comparable relative purity, supporting the development of a platform TFF process suitable for the purification of all five VP1 mRNA constructs (Figure 5C).

Several opportunities remain to further evaluate the applicability of this process to additional RNA modalities. Evaluation of additional RNA sizes could define the lower and upper molecular weight limits compatible with 50 kDa membranes while maintaining high recovery. Although membrane loadings up to 20 g/m^2^ had no measurable impact on process performance, higher loadings should be evaluated to determine practical loading limits for larger-scale manufacturing. Emerging RNA modalities, including self-amplifying RNA (saRNA) and circular RNA (circRNA), should also be evaluated because their distinct molecular architectures may influence membrane sieving behavior [29,30]. Additional mechanistic studies are also needed to better understand the relationship between RNA conformation and membrane sieving. Experimental characterization and computational analysis of mRNA contract folding could provide further insight into the molecular features responsible for the construct-dependent sieving behavior observed in this study. The optimized TFF process maintained high recovery, purity, and scalability across multiple mRNA constructs and enabled the purification of a pentavalent norovirus vaccine under a common set of operating conditions. Incorporation of RNA structural considerations into membrane selection and process design may facilitate the development of future multivalent mRNA vaccines.

## Figures and Tables

**Figure 1:**
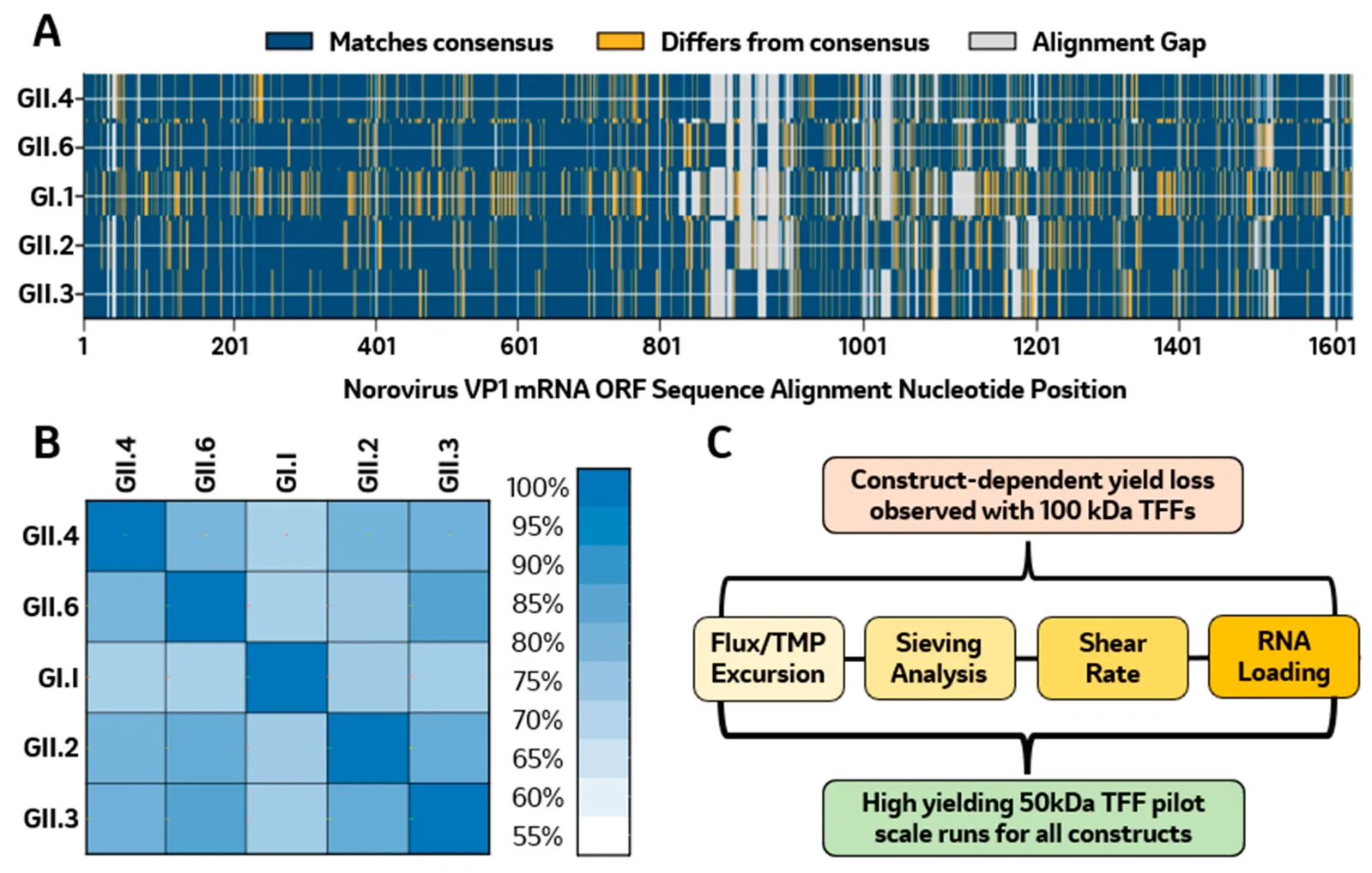
Sequence diversity and TFF process-development strategy for five norovirus VP1 mRNA constructs. (**A**) VP1 mRNA ORF sequence alignment. Positions matching the alignment consensus are shown in blue, positions differing from the consensus are shown in gold, and alignment gaps are shown in gray. (**B**) Pairwise nucleotide identity matrix derived from the VP1 ORF multiple-sequence alignment. (**C**) Overview of the TFF process-development strategy.

**Figure 2:**
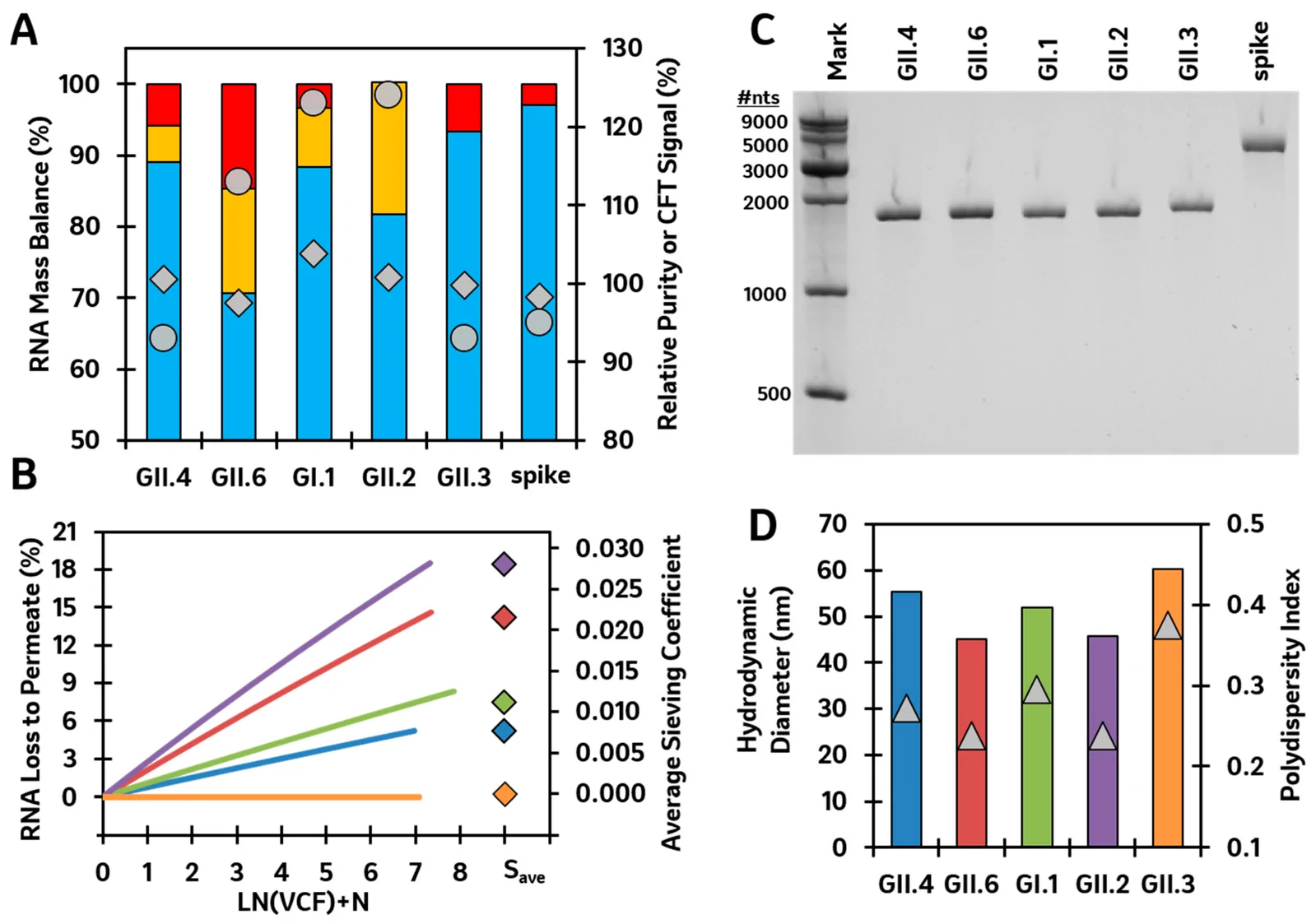
Purification of six mRNA constructs using 100 kDa hollow fiber TFF operated at a TMP of 2–3 psig, shear rate of 2000 s^−1^, and membrane loading of 4 g/m^2^. (**A**) RNA mass balance showing TFF Product Yield (blue), RNA Loss to Permeate (yellow), System Loss (red), and relative purity (grey diamonds) and relative CFT signal (grey circles) of the TFF Product. (**B**) RNA Loss to Permeate modeled as a function of average observed sieving coefficient (*S_ave_*) for GII.4 (blue), GII.6 (red), GI.1 (green), GII.2 (purple), and GII.3 (orange). (**C**) Agarose gel electrophoresis analysis of TFF Product samples and RNA marker with number of nucleotides (#nts) shown. (**D**) Dynamic light scattering (DLS) analysis of TFF Product samples showing Z-average hydrodynamic diameter (bars) and polydispersity index (grey triangles).

**Figure 3:**
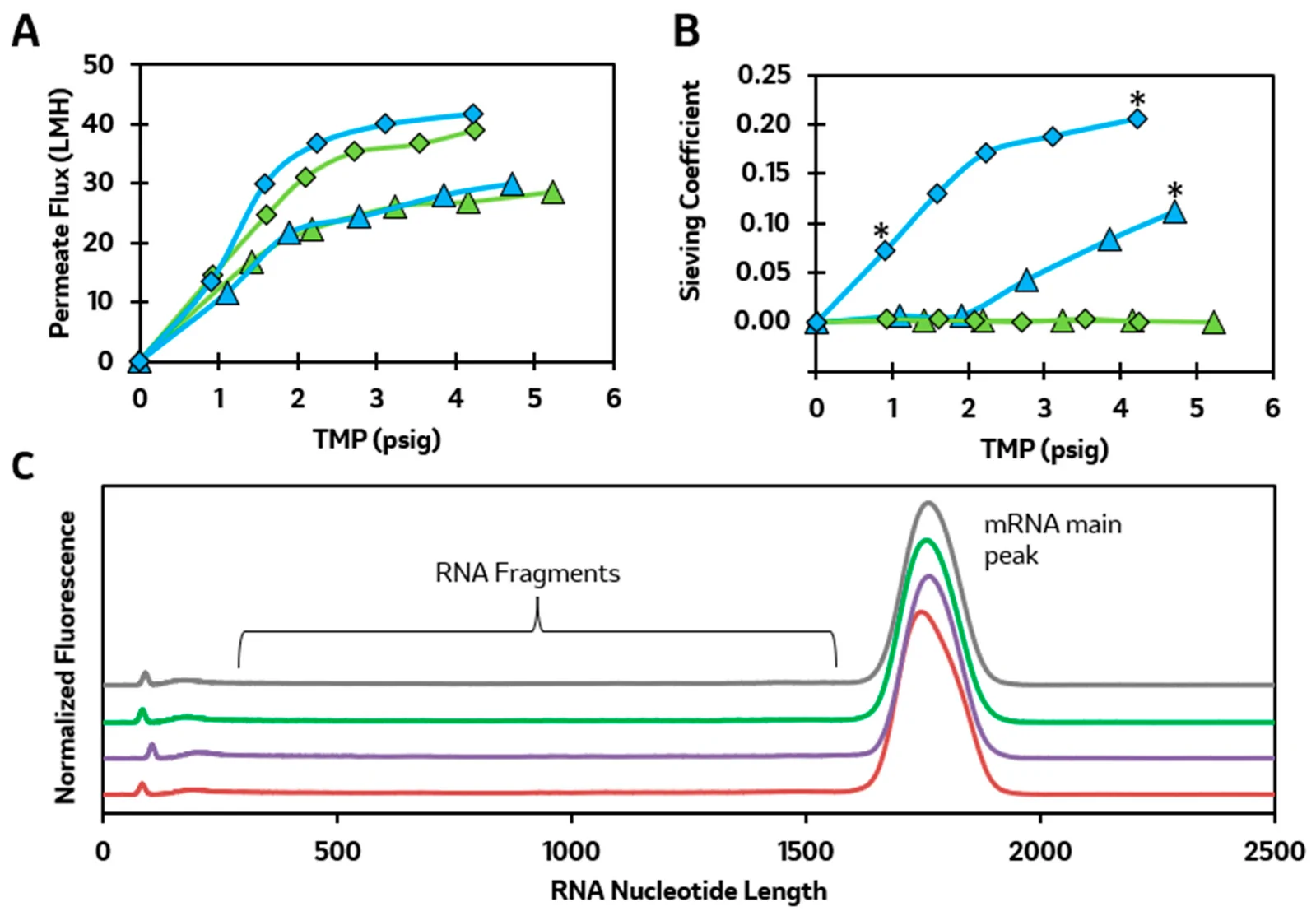
Investigation of GII.6 mRNA sieving with flux/TMP excursions and permeate CE analysis using a shear rate of 2000s^−1^ and a loading of 8 g/m^2^. (**A**) Flux/TMP excursion with 100 kDa (blue) and 50 kDa (green) TFF hollow fibers at an initial [mRNA] of ~0.5 mg/mL (diamonds) and a concentrated [mRNA] of ~2 mg/mL (triangles). (**B**) Analysis of sieving coefficients at each flux/TMP setpoint with 100 kDa (blue) and 50 kDa (green) TFF hollow fibers at an initial [mRNA] of ~0.5 mg/mL (diamonds) and a concentrated [mRNA] of ~2 mg/mL (triangles). A star indicates a permeate sample analyzed by CE in Figure 2C. (**C**) CE electropherogram analysis of 100 kDa TFF product (red) and permeate samples from a 100 kDa TFF at an initial [mRNA] of ~0.5 mg/mL and a TMP of ~1 psig (purple) and ~4 psig (green) and at a concentrated [mRNA] concentration of ~2 mg/mL and a TMP of ~5 psig (grey).

**Figure 4:**
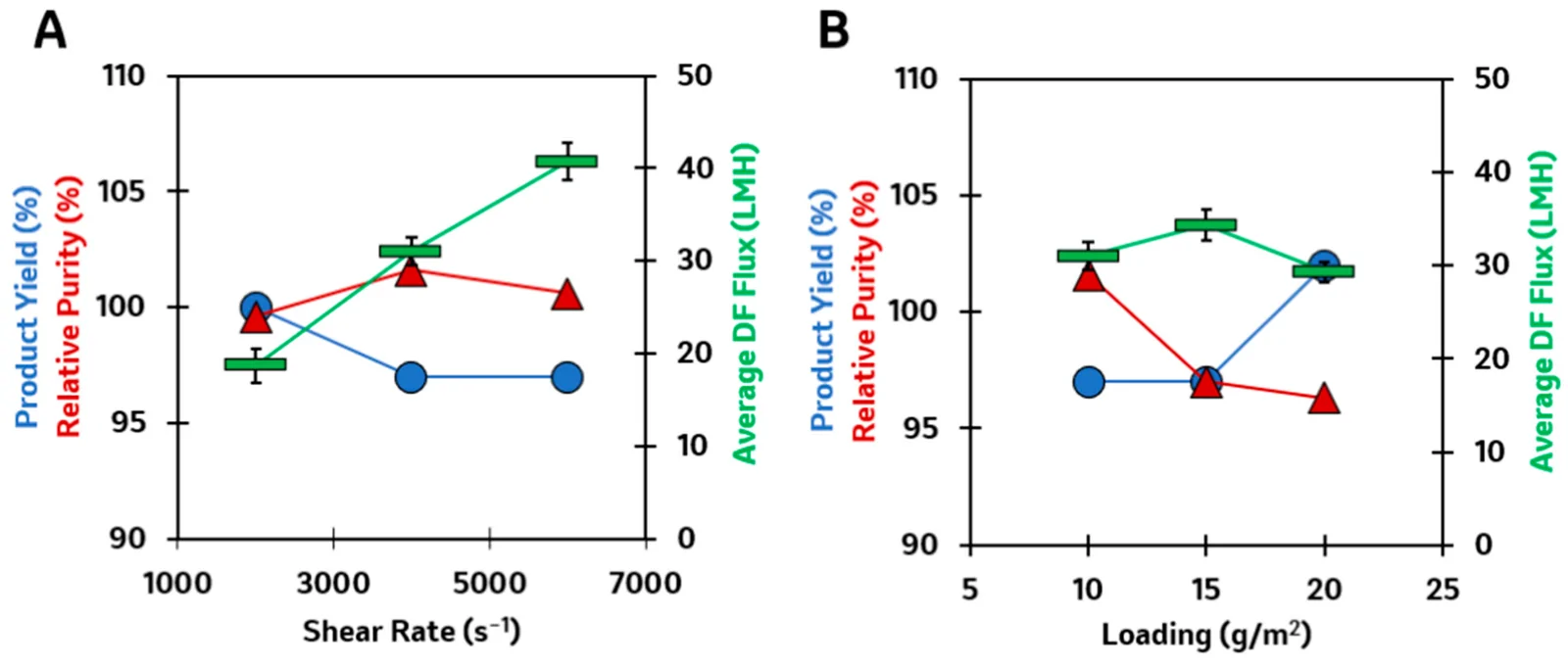
Evaluation of shear rate and loading on GII.4 purification using 50 kDa TFF and a TMP control of 2–3 psig. (**A**) Effect of shear rate on TFF product yield (blue circle), RNA relative purity (red triangle), and average diafiltration flux (green bar) using a loading of 10 g/m^2^. (**B**) Effect of RNA filter loading on TFF product yield (blue circle), RNA relative purity (red triangle), and average diafiltration flux (green bar) using a shear rate of 4000 s^−1^.

**Figure 5:**
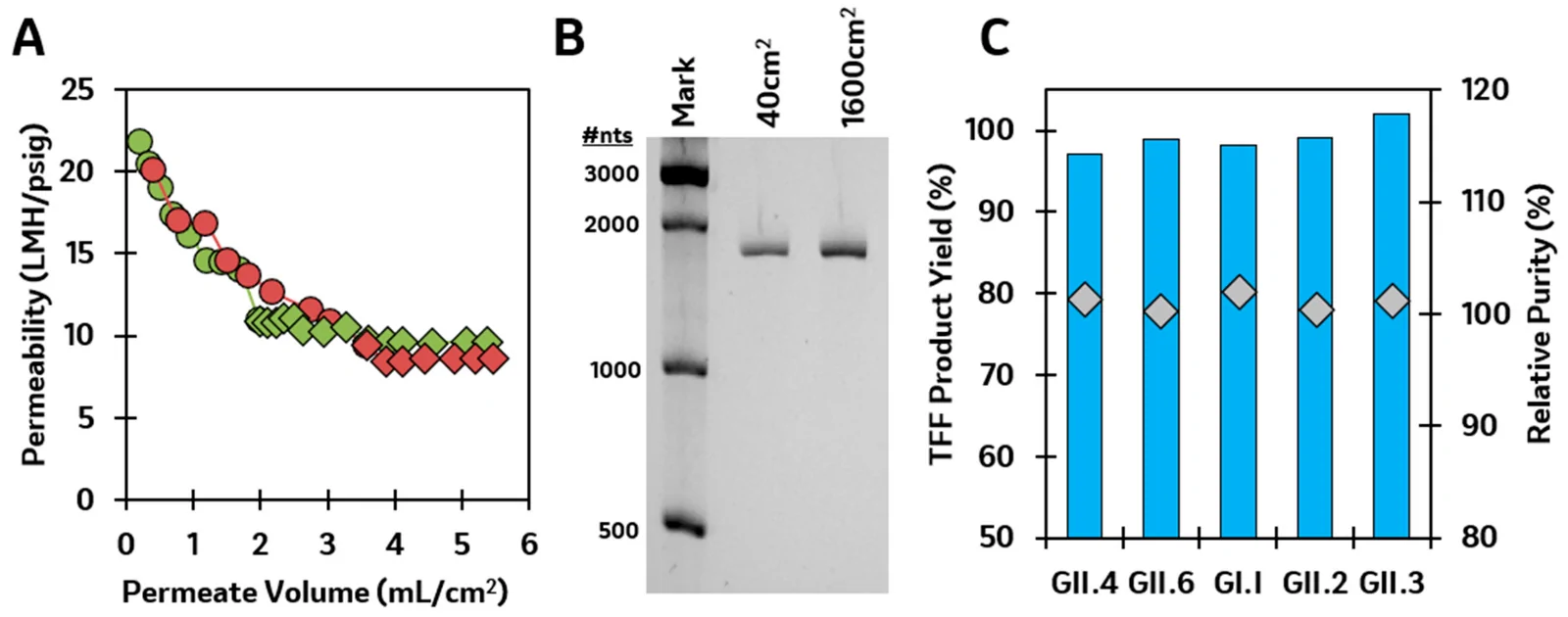
Demonstration of TFF scale-up using 50 kDa hollow-fiber membranes operated at a TMP of ~3–4 psig and a shear rate of 4000 s^−1^. (**A**) Membrane permeability during concentration (circles) and diafiltration (diamonds) for GII.4 mRNA processed in a 40 cm^2^ system (green) operated at a loading of 15 g/m^2^ and a 1600 cm^2^ system (red) operated at a loading of 12 g/m^2^. (**B**) Agarose gel electrophoresis analysis of GII.4 mRNA TFF product samples from the 40 cm^2^ and 1600 cm^2^ systems and RNA marker with number of nucleotides (#nts) shown. (**C**) TFF product yield (blue bars) and relative purity (gray diamonds) from representative pilot-scale purification runs of the five VP1 mRNA constructs using 1600 cm^2^, 50 kDa hollow-fiber membranes operated at a loading of 8–9 g/m^2^.

**Table 1:** Properties of mRNA constructs evaluated in this study.

mRNA Construct	Complete mRNA Length (nts)	Complete mRNA MW (kDa)	ORF GC Content (%)	Predicted ORF MFE (kcal/mol/100 nt)
Norovirus GII.4 VP1	1870	600	64.2	−39.5
Norovirus GII.6 VP1	1890	610	66.1	−42.5
Norovirus GI.1 VP1	1840	590	65.7	−40.8
Norovirus GII.2 VP1	1870	600	65.3	−41.7
Norovirus GII.3 VP1	1890	610	65.5	−41.5
Coronavirus Spike	3760	1210	59.0	−38.8

**Table 2:** Comparison of TFF performance at lab and pilot scale using a 4000 s^−1^ shear rate.

TFF Area (cm^2^)	Loading (g/m^2^)	Initial VCF	DF Conc (mg/mL)	DVs	Average DF Flux (LMH)	TFF Product Yield (%)	Relative Purity (%)	Relative CFT Signal (%)
40	15	5.2	3.0	5.9	31.0	97	103	Not tested
1600	12	9.3	3.0	5.4	30.7	98	101	116

## Data Availability

Relevant data in the manuscript is available upon request from the corresponding author.
